# King's College

**Published:** 1900-11-03

**Authors:** 


### KING'S COLLEGE.

### OPENING OF NEW LABORATORIES.

The fine new scientific laboratories at King's College, the result of a comprehensive scheme of extension and improvement of the teaching accommodation of the College, resolved on by the Council in 1899, are now open and in use. The whole south wing has been raised by an additional storey, and this includes the new geological, comparative anatomy, and botanical departments. The second storey of the north wing has been largely reconstructed. It contains the physiological and bacteriological departments. The fine room on the first floor has now been allotted to the architectural department. The reconstruction of the anatomical department and medical museum is also nearly complete, but the equipment is still in progress.

The necessity for increased accommodation for the physiological work has been felt for a long time. The number of students attending the advanced and special classes has steadily increased, and last session reached a total of 84. At the same time research, which has always formed a large feature in the work of the laboratory, has been carried out under difficulties. These are now removed, and both teaching and research will be able to progress side by side. For microscopic work light is, of course, essential, and this has been secured in all the rooms by a combination of side windows and skylights. Electric light, as well as gas and water, are provided throughout. All the fittings are of the latest kind, and much new apparatus has been provided. The rooms devoted mainly to students' work are a lecture theatre, shared also by the bacteriological section, and a spacious central laboratory which will seat over 100 students. The work-tables are suited either for microscopic work or for practical work in chemical physiology. There are, in addition, sixteen separate tables, provided with shafting and all the necessary electrical apparatus, for the study of experimental physiology. In addition to this, there are four rooms devoted to research; a large room for investigations in chemical physiology; another, admirably fitted, for experimental physiology; a dark room for photographic and galvanometer work; and a private room for the professor; as well as the necessary store-rooms and accommodation for the laboratory attendants.

By the provision of the new physiological laboratory the old one has become available for increased accommodation in the anatomical department. The dissecting room, which was previously far too small, has been nearly doubled in size, and all the accessory rooms necessary in a well-equipped anatomical department are now provided. The section of the College Museum which relates to pathology will also be housed in part of the old physiological rooms in the basement, and a new and very handsome room, near the dissecting room, has been built for the anatomical portion of the museum.

King's College was the pioneer in England in providing laboratories (in 1886) devoted to bacteriology, and continues to hold a unique position in giving systematic technical instruction in this special branch of medical and scientific education to medical men in practice, officers of health, colonial and foreign practitioners, veterinary surgeons, agriculturists and analysts, whether previously connected with the College or not, and the subject has recently been added to the course of training for students of the Medical School and of the Institute of Chemistry. There is now a very large class-room devoted to the technical education of post-graduate and other students from all parts of the world. Every student, with his own hands, goes through the whole practical course, and is further assisted by lectures and practical demonstrations. About 1,500 students have worked in this laboratory, and several have been specially trained with a view to investigating plague, cholera, yellow fever, madura, and other tropical diseases, as well as the diseases of farm stock which are prevalent in our colonies and in foreign countries. In the technical laboratory research work has been undertaken for the Board of Agriculture and colonial governments, and analytical researches are undertaken for county councils and other local governing bodies. The research room and library is used by advanced students and by the professor. Any overlapping of classes or overcrowding of students is now prevented, and senior students can work undisturbed. A new feature is the bacteriological library of about 1,000 volumes and pamphlets, lent by the professor for the use of the senior students. The library is unique and of the greatest value in providing works of reference, as well as the standard text-books ready at hand for those studying the subject or undertaking original research.

A lecture theatre has been built with the usual equipment, including an electric lantern and screen. The theatre is jointly for the use of the bacteriological and physiological departments, and will accommodate about 200 students.

The principal collections used in teaching geology, comparative anatomy, botany, and materia medica are now housed in the second floor north corridor, where there is plenty of light. A few specimens are exhibited in glass cases, but most are contained in cabinets beneath; while the adjoining geological and botanical laboratories contain additional collections. On the east side of the corridor the preparations are all recent, and chiefly refer to comparative anatomy. On the west side the cases hold specimens of rock-forming minerals, types of rocks, ores of common metals, and fossils. On the walls are casts, skulls, and stuffed specimens. Botany is represented by some remarkably fine and complete collections.

The general geological laboratory and lecture room will accommodate 50 students. It is fitted also for practical work, gas, water and electric light being laid on. The materia medica and pharmacological collection of specimens used in teaching is contained in the upper part of the corridor, and is open for purposes of study: the lectures are given in another part of the building.

In physics, the Wheatstone laboratory is well equipped, and the physical museum contains a fine collection of historical and experimental apparatus.

On Tuesday afternoon (30th inst.) the laboratories were formally opened by Lord Lister, P.R.S., who was supported by the Lord Mayor and sheriffs, and the Council and professional staff of the college. The audience was a distinguished and representative one.

The Principal (Rev. A. Robertson), in introducing Lord Lister, said the pleasure felt by the staff at meeting his lordship and so distinguished a company was tinged with a touch of sorrow caused by the loss of a brilliant young life—that of Prince Victor—by the Queen and by the nation.

Lord Lister said it must perhaps seem strange that so large a gathering of distinguished men should have come to witness the opening of certain laboratories; but reflection would show that the occasion was not unworthy of such celebration. It was an occurrence which marked a step in the higher education of the metropolis. It had long been felt by the Council that more adequate provision for practical instruction was imperatively necessary. In some branches King's College had for long been well equipped; the Wheatstone collection was as fine a one as any existing. Physics was well provided for; and of chemistry and some other departments the same must be said—but not of all. Thus, the dissecting room was by no means adequate; the accommodation for the proper teaching of physiology was simply miserable; and in less degrees, perhaps, there were deficiencies in other departments. The Council had long been aware of these deficiencies and desired to remedy them; and they had now provided laboratories which, he felt sure, after they had inspected them, they would admit were highly satisfactory. The physiological laboratories were now, he believed, second to none in the country. Although they had not yet been formally opened, they had been for some time in use under Professor Halliburton, and had been doing good work. They had all learned with regret that Professor Hughes had returned from South Africa invalided with that insidious disease enteric fever. Happily there was less cause for anxiety as to his condition than there had been, and they all hoped soon to see him again superintending his work there. In the past their bacteriological laboratory had attracted students from all parts of the world; but they had now made important additions to it, and it no longer consisted of one room very inadequately adapted to the purposes of research. They had a fine room for that purpose now added, and they had also constructed a fine class-room, which would greatly facilitate instruction on practical bacteriology. They had all watched, with interest and with anxiety, the efforts which had been made to grapple with the cases of plague at Glasgow, and shared in the sense of relief at the success of those efforts. There must also have been a sigh of relief when it was found that the suspected case in London turned out not to be one of plague. And they would all realise that the combating and the removal of that danger in Glasgow, and that scare in London, were due entirely to the kind of research practicable in such laboratories as had now been provided. The geological and architectural and other departments would all benefit by the new laboratories, and King's College must now be said to be abreast of the age in the teaching of science in all its branches. It was a happy coincidence that at the same time that King's College was opening its new laboratories it was just entering on its new career as part of the reconstructed University of London. Personally, he should have preferred that the London University should have retained its position as an Imperial examining body, the teaching being carried on apart. But since other views had prevailed, he cordially wished success to the compromise which had been decided upon, and he hoped that all would now join to work for the common good.

The Lord Mayor (Sir Alfred Newton) briefly moved a vote of thanks to Lord Lister for his presence there, and for his address.

This was seconded by the Hon. W. F. D. Smith, M.P., treasurer of the College, who said he felt that the attendance of such a gathering was a justification for the course taken by the Council in incurring, not without anxious deliberation, an expenditure of some £20,000, to the obtaining of a considerable portion of which they could not see their way. They had only two alternatives: to incur that expenditure and so saddle themselves with a debt which they hoped would be but temporary; or to sacrifice the reputation of the College by letting it fall behind. That, they were sure, would not be temporary, but permament. So far, they had only obtained about one-fifth of the amount required, and they had to face the somewhat alarming figure of £16,000. But he hoped there might be some patriotic Londoner, who, stimulated by those proceedings, and by Lord Lister's remarks, would relieve King's College of that debt.

The vote of thanks was carried *nem con.*, and Lord Lister then formally declared the new laboratories open.

